# Comparison of the Efficiency of Two Novel Guided Bone Regeneration Devices in the Rabbit Calvarial Model

**DOI:** 10.1155/2020/8846285

**Published:** 2020-11-26

**Authors:** Osama Zakaria

**Affiliations:** Department of Biomedical Dental Sciences, Oral and Maxillofacial Division, College of Dentistry, Imam Abdulrahman Bin Faisal University, P.O. Box 1982, 31441 Dammam, Saudi Arabia

## Abstract

**Background:**

Creating a secluded large space using guided bone regeneration (GBR) is a novel osteogenesis technique used in the prevention of premature membrane exposure complications. However, this technique is not considered clinically feasible.

**Objectives:**

This study aimed to compare the outcome of the insertion of two novel GBR devices in a rabbit calvarial model in terms of mode of action, simplicity, and amount of new space and bone gained.

**Materials and Methods:**

The expansible GBR (EGBR) device, composed mainly of a titanium plate, silicone membrane, and activation screw, was inserted beneath the periosteum in the calvarial area of eight rabbits. The smart GBR (SGBR) device, composed of silicone sheets and Nitinol strips, were inserted beneath the periosteum in the calvarial area of another 10 rabbits. Half of each group was sacrificed 2 months after surgery, and the other half was sacrificed after 4 months.

**Results:**

Histological and microradiographical analysis showed that, at 2 months, the EGBR device achieved a mean space gain of 207.2 mm^3^, a mean bone volume of 68.2 mm^3^, and a mean maximum bone height of 1.9 mm. Values for the same parameters at 4 months were 202.1 mm^3^, 70.3 mm^3^, and 1.6 mm, respectively. The SGBR device had significantly higher (*P* < 0.05) mean space gain (238.2 mm^3^; 239.5 mm^3^), bone volume (112.9 mm^3^, 107.7 mm^3^), and bone height (2.7 mm; 2.6 mm) than the EGBR device at 2 and 4 months, respectively.

**Conclusion:**

Both devices proved to be effective in augmenting bone vertically through the application of GBR and soft tissue expansion processes. However, the SGBR device was more efficient in terms of mode of action, simplicity, and amount of bone created in the new space.

## 1. Introduction

Guided bone regeneration is a dental surgical technique that utilizes membranes to direct new bone regeneration at sites of deficiency in jaws, meanwhile excluding soft tissue growth. Consequently, this improves function, esthetics, or prosthetic restorations.

However, the creation of sizeable spaces is not clinically feasible [1]. In GBR, it is crucial to ensure that the soft tissue is very well managed to minimize the likelihood of premature membrane exposure complications and subsequent bacterial contamination. In most cases, contamination occurs due to insufficient soft tissue coverage during flap closure, resulting in excessive tension of tissues [2, 3]. The GBR technique has been applied in previous studies with silicone domes for bone augmentation in a rabbit model [4]. However, this technique does not allow the creation of sufficiently large spaces, which has limited its clinical applicability due to the lack of soft tissue coverage. When it is difficult to ensure primary soft tissue closure, latent exposure of the membrane typically follows [5].

Gradual periosteal lifting is a technique used to enlarge the interface over the bone surface and lifts the periosteum slowly to induce supraosseous neogenesis. This technique was first introduced by Schmidt et al. in 2002 [6]. There is evidence from animal studies that periosteal distraction has been effective at inducing osteogenesis to distract the soft tissue [7–11]. However, a major drawback to this technique is that the distraction space created has poor bone quality and soft tissue [8–12].

Expansible GBR (EGBR) is a combination of two procedures: GBR and periosteal distraction. Similar to periosteal distraction, a plate is elevated from one side by an activation screw while the gradually created space is kept isolated from the overlying soft tissue by a silicone membrane during and after device activation [12].

The smart GBR (SGBR) device is similar to the EGBR device and grows gradually into a dome shape, adopting both periosteal distraction and GBR principles. Unlike the EGBR device, it is a form of a bone generation device that has a fully automated memory-based shape, and thus, for activation and insertion to become completely concealed, only one surgical intervention is required under the periosteum. There are two ultrathin silicon sheets, between which two thin nickel-titanium (Ni-Ti) strips are implanted. Once it is activated, the SGBR device expands the overlying periosteum and soft tissue to create a separate growing space where the new bone is generated, which ensures that the soft tissue is not invaded [13]. In this study, the outcome of the EGBR and SGBR devices in a rabbit calvarial model are compared in terms of mode of action, simplicity, and volume of new space and bone gained.

## 2. Materials and Methods

The present study compared, in a rabbit calvarial model, the efficacy of two devices (SGBR and EGBR) in the generation of bone using the GBR principle. Eighteen Japanese male white rabbits with a weight range of 2.5 to 3 kg were included in this comparative study. The study protocol was approved by the Committee of Animal Experiments at Tokyo Medical and Dental University (Approval No. 01202241A). Surgical and tissue analysis phases of the study were performed at Tokyo Medical and Dental University (Japan).

Data were retrieved from two groups. Animals in Group 1 (*n* = 8) received the EGBR device, whereas those in Group 2 (*n* = 10) received the SGBR device. For each group, half of the animals were sacrificed after a 2-month consolidation period, and the remaining half were sacrificed after a 4-month consolidation period.

**Figure 1:**
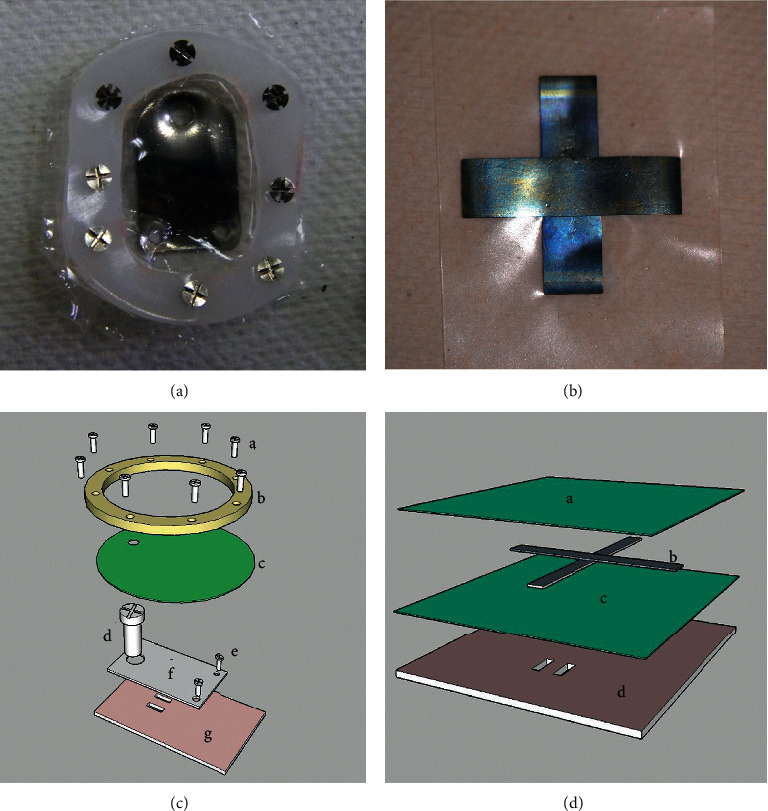
Photograph of (a) the expansible guided bone regeneration (EGBR) device and (b) the smart guided bone regeneration (SGBR) device. (c) Illustration showing the six components of the EGBR device: (g) original bone, (f) rectangular shape titanium elevating plate (16 mm × 10 mm × 0.5 mm), (e) titanium fixation miniscrews (3 mm in length and 1 mm in diameter), (d) titanium elevating screw (5 mm in length and 2 mm in diameter), (b) plastic ring (24 mm × 18 mm × 0.5 mm external dimensions) with eight equidistant holes for (a) fixation screws. The (c) silicone membrane (0.05 mm thick) contains one hole for the activation screw (1.8 mm wide). (d) Illustration showing the three components of the SGBR device: (a, c) two silicone sheets (22 mm width, 22 mm length, and 0.25 mm thickness), and (b) two Ni-Ti strips (14 mm length, 2.5 mm width, and 0.1 mm thickness) placed perpendicular to each other (d) original bone.

Construction of the EGBR and SGBR devices is illustrated in Figures 1(c) and 1(d).

### 2.1. Surgery

All animals underwent similar surgical procedures. General anesthesia was achieved through preoperative intramuscular ketamine (50 mg/kg Ketalar; Sankyo, Tokyo, Japan) and thiopental sodium (25 mg/kg Ravonal; Tanabe, Tokyo, Japan). In addition, 1.8 mL (2% xylocaine/epinephrine 1 : 80,000; Dentsply Sankin, Tokyo, Japan) was administered as a local anesthetic at each insertion site before surgery. Aseptic conditions were maintained in all operations. Animals were randomly assigned to receive one of the guided bone regenerations devices using a coin toss method.

### 2.2. EGBR Device Surgical Protocol

**Figure 2:**
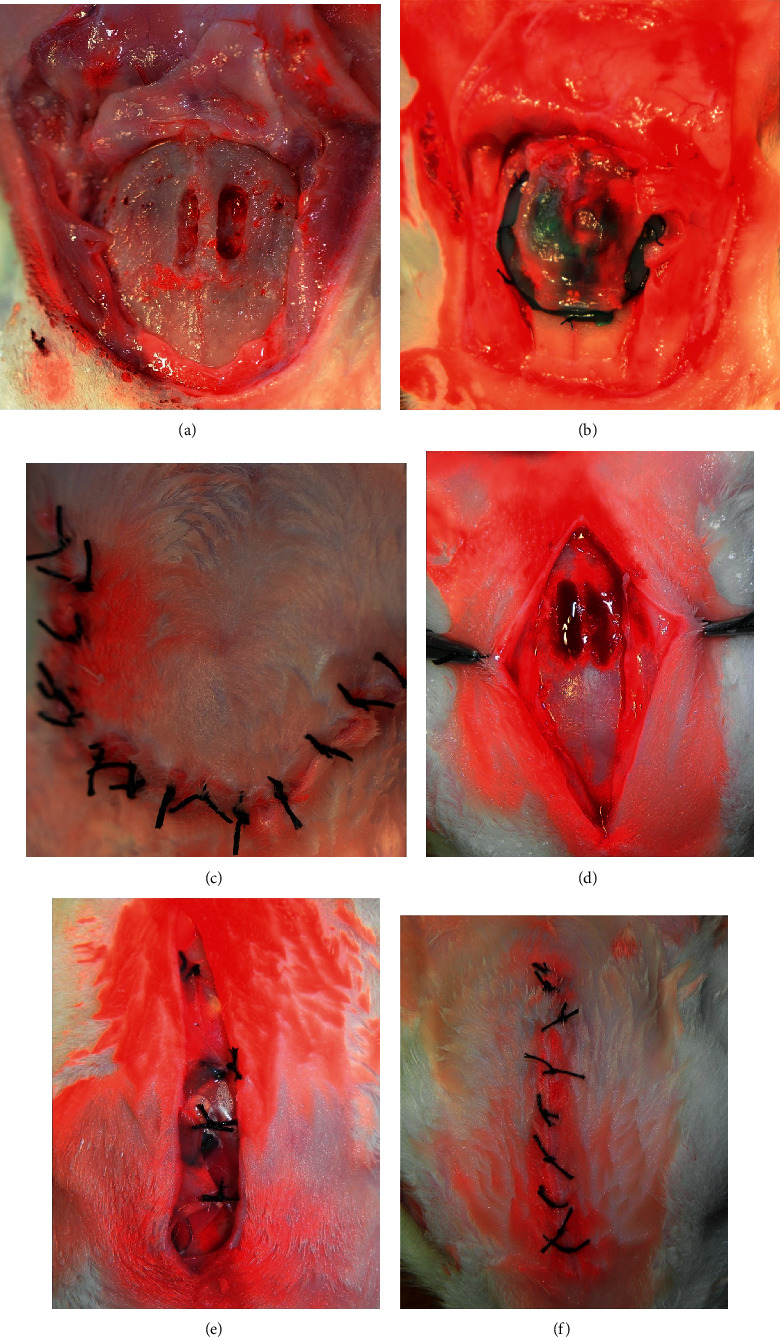
Images of the surgical procedures. For the expansible guided bone regeneration (EGBR) device, (a) periosteal and skin U-shaped flaps are reflected and the cortical groove is created. (b) The EGBR device is fixed over the calvarium and covered by the periosteum. (c) The skin flap is sutured back in place. For the smart guided bone regeneration (SGBR) device, (d) the midline incision is made, skin and periosteal flaps are reflected, and the cortical groove is created. (e) The SGBR device is fixed over the calvarium and covered by the periosteum. (f) The skin flap is sutured back in place.

**Figure 3:**
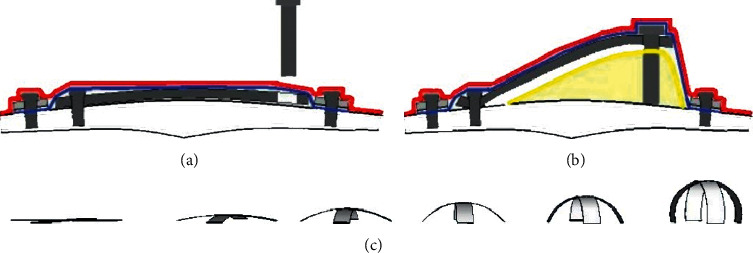
Illustration showing the mode of action of the expansible guided bone regeneration (EGBR) and smart guided bone regeneration (SGBR) devices. (a) The EGBR device is inserted in the calvarium site without activation. (b) The activation screw is inserted 1 week after EGBR device insertion. (c) The SGBR device is inserted in a flat unactivated state, and then, it becomes activated to maximum designed size.

A subperiosteal incision and U-shaped skin incision were performed to expose the calvarial bone. The skin flap was reflected, followed by the periosteum flap. Under irrigation with saline, the occipital bone was decorticated to form a groove 10 × 3 mm (Figure 2(a)). A plate was elevated from the activated area and was connected to the bone surface using two mini screws from one of its ends (Figure 1(c)). The silicone membrane was secured by the plastic ring and placed such that it covered the elevated plate, and then, eight microscrews were used to fix the ring to the calvarial bone (Figure 1(c)). The skin flap was sutured back in layers (Figure 2(c)). After one week, an incision of soft tissue was carried out over the screw hole of the elevated plate. The screw was fixed inside the hole to connect to the elevated plate. The screw was rotated at an angle of 360°, which raised the titanium elevating plate by 1 mm and subsequently moved both the overlying silicone membrane and soft tissue higher. Activation to the plate was applied at a rate of 1 mm/day for 5 days, and this caused the overlying silicone membrane to increase slowly until it became tent shaped (Figures 3(a) and 3(b)).

### 2.3. SGBR Device Surgical Protocol

Subperiosteal incision and a midline skin incision were performed to expose the calvarial bone. The skin flap was reflected, followed by the periosteum flap. Under irrigation with saline, the occipital bone was decorticated using a No. 4 round bur to form a groove 10 × 3 mm (Figure 2(d)). The occipital bone contained an exposed area containing the grooves, where the device was placed, and pressure was applied at the center using a blunt object. When the skin flaps and periosteal flaps were sutured back, the pressure was released. One side of the membrane, which is active, resulted in a slight skin elevation, but the flap was not exposed (Figures 2(e) and 2(f)).

The animals used for this study were fed using standard laboratory water and food and stayed in a standard cage inside the animal experimental room throughout the study. Later, they were sacrificed by an extra dose of thiopental sodium. In each experiment, half of the total number of animals (*n* = 4 in EGBR and *n* = 5 in SGBR) were sacrificed at each time point (2 and 4 months). The cranial bone remained separate from the animal head for two weeks in neutral-buffered 10% formalin until it was fixed.

### 2.4. Microcomputed Tomography Analysis

The specimens were fixed using formalin and then scanned using a high-resolution microcomputed tomography (micro-CT) imaging system (SMX-90CT; Shimadzu, Kyoto, Japan) and was gradually increased to 60 *μ*m under a current of 60 *μ*A and applied voltage of 75 kV.

A total of 10 serial sagittal images were derived for each of the scanned specimen (1 image/mm). The brightness and contrast of the images were adjusted automatically and later converted into 8-bit grayscale before recording the measurement. Image analysis software (ImageJ version 1.47, NIH, Bethesda, MD) was used to analyze the images to obtain the maximum heights of the new bone, newly created space, and volume of new bone tissue volume [13].

### 2.5. Histological Processing

The samples were dehydrated after calvarial bone fixation using ethanol at gradual concentrations and embedded in resin (Technovit 7,200; Heraeus Kulzer GmbH, Wehrheim, Germany). Sections were cut (Exakt, Mesmer, OstbEinbeck, Germany) and then ground to obtain a smooth sample of approximately 40–50 *μ*m thickness. Once the required smoothness was achieved, the sections were stained using 0.1% toluidine blue (Sigma-Aldrich). A BZ-8000 microscope (Keyence, Osaka, Japan) was used to perform histological examinations. The data obtained were analyzed using BZ-Analyzer software (Keyence).

### 2.6. Statistical Analysis

Statistical analysis was carried out in SPSS version 17.0 software (SPSS Inc., Chicago, IL, USA). Normality of the data was analyzed using the Shapiro–Wilk test. Means and standard deviation were reported for each group, and *t*-tests were used to compare the means of new bone height, newly created space, and the volume of new bone tissue between groups. The statistical significance threshold was set at *P* < 0.05.

## 3. Results

Following implantation of the EGBR or SGBR devices, all animals resumed normal dietary habits once they had recovered from general anesthesia. No animals developed infections or inflammation. The devices remained intact on the calvarium, and they were not displaced or exposed during the experimental period.

### 3.1. Microradiographical Findings

**Figure 4:**
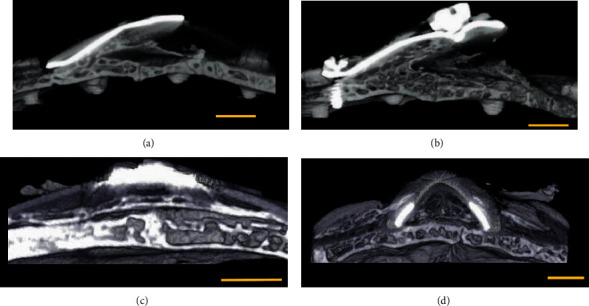
Longitudinal microradiograph section of activated expansible guided bone regeneration (EGBR) and smart guided bone regeneration (SGBR) devices at the 2- and 4-month time points. (a) The EGBR device at the 2-month time point where one side is elevated, and new bone is formed in the space below, atop of the original bone surface. (b) The EGBR device at the 4-month time point shows almost the same microradiographical picture as at the 2-month time point; however, the bone is more abundant. (c) The SGBR device at the 2-month time point where a new layer of bone is formed above the original bone below the silicone sheet. (d) The SGBR device at the 4-month time point shows a similar picture with more abundant bone formation. Scale bar = 4 mm.

All examined calvaria-like bone samples showed significant new bone formation. By the 2-month time point, coronal sections of the areas created below the devices showed a nonsymmetrical triangular shape that was partly filled with new bone tissue (Figure 4(a)). SGBR coronal sections showed a segment shape that was also partly filled with new bone tissue (Figure 4(c)).

The 4-month time point showed a similar radiographic picture to a 2-month time point for both the EGBR and SGBR devices. However, at 4 months, the new bone formation in both devices showed a radiopacity and thickness comparable to the original bone (Figures 4(b) and 4(d)).

Radiomorphometric data on the size of space created by each device, amount of bone gained, and the maximum bone height attained in the samples from animals from each group (both devices, at both time points) are summarized as mean and standard deviation in Table 1.

### 3.2. Histological Findings

**Figure 5:**
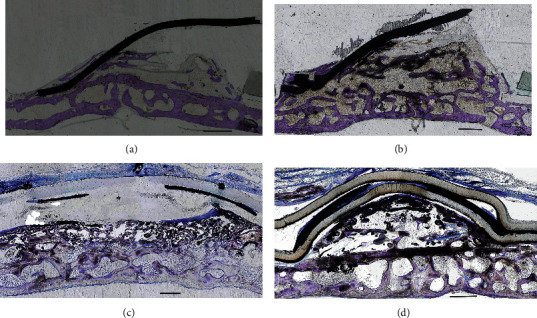
Histological longitudinal sections of the calvaria with toluidine blue staining of the original bone and newly created space after device insertion. (a) Expansible guided bone regeneration (EGBR) device, 2-month group; the new space is occupied by newly generated bone with a space on the top. (b) EGBR device, 4-month group. (c) Smart guided bone regeneration (SGBR) device, 2-month group; the new space is partially occupied by newly-generated bone with a space on the top. (d) SGBR device, 4-month group; more bone formation is visible than in the 2-month group. Scale bar = 1 mm.

The results showed that, in all specimens, the calvarial bone showed marrow cavities surrounded by mineralized bone with double-thick and compact layers where the spaces between the trabeculae were filled with connective tissue (Figures 5(a)–5(d)).

At the 2-month time point, microscopic examination of specimens from the EGBR device group showed that the space was almost filled by the newly generated bone tissue. A sizeable amount of intramembranous bone trabeculae was used to cover below the silicone membrane and titanium plate. Blood vessels and fat marrow filled the interspace between the trabeculae. In addition, a cortical bone plate had formed above the new bone tissue (Figure 5(a)).

At the 2-month time point, microscopic examination of specimens from the SGBR device group indicated that the new bone tissues occupied a large proportion of the newly created space. The area below the silicone membrane was covered by a sizeable amount of intramembranous bone trabeculae (Figure 5(c)). At the 4-month time point, histological images were compared to those of the 2-month group (Figure 5(d)).

There was a remarkable increase in bone trabeculae thickness, at the same time as a gradual decrease in the intervening vascular connective tissue, in both the 2- and 4-month groups (Figure 5(a)–5(d)). In EGBR specimens, it was noted that there was a tendency of the bone trabeculae to increase alongside the inside surface of the silicone membrane and titanium plate (Figure 5(a)). In SGBR specimens, the new trabecular bone was observed lining the inner wall of the silicon membrane (Figure 5(d)).

In all EGBR specimens, the plastic ring tightened below the silicone ring against the original bone, and as a result, there was no gap created between the calvaria and the device (Figures 5(a) and 5(b)), while in all SGBR animals, it was observed that the silicone membrane peripheries were very close to the original bone, and as a result, there was no tissue or space in between (Figures 5(c) and 5(d)). In all specimens, there was an unoccupied space above the newly formed tissues (Figures 5(a)–5(d)).

## 4. Discussion

Histological and microradiographical results confirmed that both devices successfully created a dome-shaped space in the rabbit calvarial model. The recorded volumes by the two devices exceed those reported in past GBR studies [13–15]. Notably, the mean space gained by the SGBR device was 14% higher than that of the EGBR device at the 2-month time point while the same parameter was 18% higher at the 4-month time point.

The results showed that the newly created spaces were occupied by new bone. The SGBR devices showed better results compared to the EGBR devices in terms of the amount of new bone formation per volume created, with 47% at 2 months and 44% at 4 months, whereas EGBR values ranged from 32% at 2 months to 34% at 4 months postoperatively.

However, the main difference between the two devices was in the mode of action. While the EGBR devices depended on advancing a screw in the elevating plate to gain the space gradually, the SGBR device used the body heat sensitivity of the Ni-Ti strips for the same purpose. Moreover, the EGBR device needed a second surgical procedure to insert the activation screw, one week after the first surgery.

A further advantage of the SGBR device was its simplicity: it required fewer components than the eight components required for the assembly of a single EGBR device. The larger number of components in the EGBR device was reflected in the increased complexity and increased duration of surgical procedures, which may increase suffering in the animal.

The EGBR proved occlusive against overlying soft tissue by the aid of miniscrews to keep the silicone membrane close to the bone surface (Figure 1). In contrast, in the SGBR device, the same function was achieved only by extending the silicone membrane for 5 mm all around the confines of the Ni-Ti strips, so that the excess silicon acted to keep the soft tissue away from the newly created bone inside the new space.

The EGBR device showed some soft tissue leakage at the penetration point of the elevation screw through the circular hole in the membrane, due to tension on the silicone membrane. This drawback was not observed with the SGBR device because the silicone membrane was completely intact throughout the experiment as there was no need for an activation screw. Both the EGBR and SGBR devices showed high stability during the experiment. While the EGBR device needed eight screws to achieve this goal, the SGBR device required only the pressure of the overlying soft tissue for stability.

## 5. Conclusions

The SGBR was observed to be more effective and efficient than the EGBR device. This may be due to the self-activating mechanism of the device, which kept the newly generated bone secured from disruption throughout the experiment.

## 6. Recommendation

Further investigation of the efficiency of the SGBR device is warranted in a higher animal model.

## Figures and Tables

**Table 1:** Space, bone volumes, and bone height values for each device at 2 and 4 months.

		2 months				4 months			
		EGBR *n* = 4	SGBR *n* = 5	*t*	*P*	EGBR *n* = 4	SGBR *n* = 5	*t*	*P*
Space volume (mm^3^)		207.15 ± 31.76	238.25 ± 20.08	1.81	0.11	202.13 ± 21.72	239.5 ± 18.2	2.82	0.026^*∗*^
vs. 4 months	*t*	0.26	0.10						
	*P*	0.81	0.92						
Bone volume (mm^3^)		68.2 ± 22	112.86 ± 19	3.27	0.014^*∗*^	70.3 ± 14	107.76 ± 17	3.54	0.0095^*∗*^
vs. 4 months	*t*	0.16	0.45						
	*P*	0.88	0.67						
Bone height (mm)		1.85 ± 0.46	2.7 ± 0.35	3.16	0.016^*∗*^	1.6 ± 0.43	2.6 ± 0.36	3.81	0.0067^*∗*^
vs. 4 months	*t*	0.79	0.45						
	*P*	0.46	0.67						

*∗*Significant difference between groups in a *t*-test, where *P* < 0.05. EGBR, expansible guided bone regeneration; SGBR, smart guided bone regeneration.

## Data Availability

The micro-CT scanning data used to support the findings of this study were supplied by Osama Zakaria under license and so cannot be made freely available. Requests for access to these data should be made to oazakaria@iau.edu.sa (Osama Zakaria).
